# Insertion vélamenteuse du cordon

**DOI:** 10.11604/pamj.2014.19.55.4487

**Published:** 2014-09-23

**Authors:** Mehdi Kehila, Sobhi Kdous

**Affiliations:** 1 Faculté de Médecine de Tunis, Service C du Centre de Maternité et de Néonatologie de Tunis, Tunisie

**Keywords:** Insertion vélamenteuse, cordon, échographie obstétricale, Velamentous insertion, cord, obstetric ultrasound

## Image en medicine

**Figure 1:**
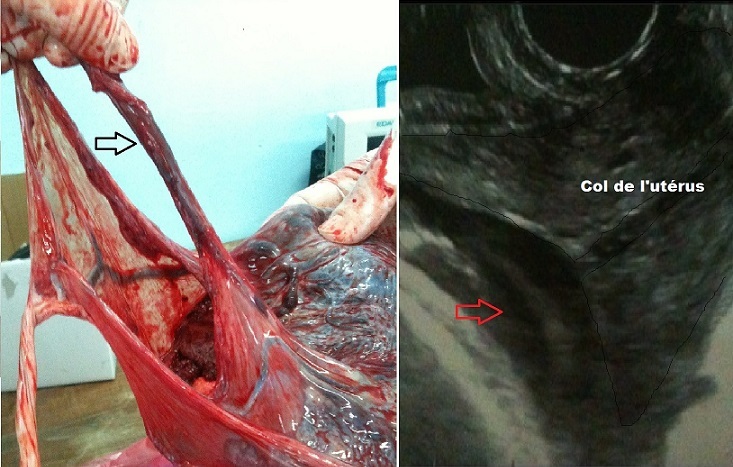
Insertion vélamenteuse du cordon: un vaisseau praevia (flèche noire) et sa traduction échographique (flèche rouge)

Il s'agit d'une patiente âgée de 36 ans, sans antécédents particuliers, G3P3, ayant accouché à deux reprises par voie basse. Elle a été admise par le biais des urgences à un terme de 38 Semaines d'aménorrhée pour entrée spontanée en travail. Une échographie obstétricale a été pratiquée objectivant un placenta antérieur loin du col et une image oblongue rappelant le cordon ombilical interposée entre la présentation et l'orifice interne du col (Flèche). Le diagnostic de procubitus du cordon a été suspecté et une césarienne en urgence a été décidée. Lors du transfert au bloc opératoire, une rupture spontanée des membranes est survenue. La césarienne s'est déroulée en extrême urgence sous anesthésie générale permettant l'extraction d'un nouveau-né avec un score d'Apgar à 8 à 1 min puis 10 à 5 minutes. L'examen du délivre a objectivé une insertion placentaire antérieure loin du col avec insertion vélamenteuse du cordon et de gros vaisseaux qui partent du point d'insertion du cordon et qui parcourent la surface des membranes (Figure 1).

